# The Interaction Among Depressive Symptoms, Pain, and Frailty in Middle-Aged and Older Adults: A Longitudinal Cross-Lagged Panel Analysis

**DOI:** 10.1155/da/5566680

**Published:** 2025-09-09

**Authors:** Tian-Ming Song, Xue Chen, Xue-He Chen, Si-Jia Tan, Chen-Huan Song, Lin Fan, Jia-Yi Li, Hong-Li Li

**Affiliations:** ^1^School of Nursing, China Medical University, Shenyang, Liaoning Province, China; ^2^Department of Psychiatry, The Fourth Affiliated Hospital of China Medical University, Shenyang, Liaoning Province, China; ^3^Department of Neurosurgery, Lianyungang No. 1 People's Hospital, Lianyungang, Jiangsu Province, China; ^4^Department of Operating Room, Lianyungang No. 1 People's Hospital, Lianyungang, Jiangsu Province, China

**Keywords:** cross-lagged panel models, depressive symptoms, frailty, pain, random intercept cross-lagged panel models

## Abstract

**Background:** Depressive symptoms, pain, and frailty interactions in middle-aged and older adults do have longitudinal research support, yet the currently available evidence remains insufficient for a comprehensive understanding. This study aimed to examine their interrelationships and underlying mechanisms.

**Methods:** This study utilized data from the China Health and Retirement Longitudinal Study (CHARLS), which includes four assessments for depressive symptoms, pain, and frailty over 7 years. We included 4961 participants aged 45 years and older in our analysis. We employed cross-lagged panel models (CLPMs) and random intercept CLPMs (RI-CLPMs) to analyze the bidirectional temporal relationships at the between-person and within-person levels.

**Results:** Cross-lagged panel analysis revealed bidirectional associations between depressive symptoms and pain. A reciprocal predictive relationship was also identified between frailty and pain. After controlling individual differences with the RI-CLPM, depressive symptoms continued to positively predict pain during subsequent periods. However, the predictive effect of pain on subsequent depressive symptoms turned nonsignificant. Although a cross-lagged relationship remained between pain and frailty, it showed a declining trend.

**Limitations:** While engagement in depressive symptoms, pain, and frailty was assessed via questionnaire, long measurement intervals may not capture short-term fluctuations in the state of each variable.

**Conclusions:** This study differentiated within- and between-individual effects, uncovering distinct lagged effects of pain, depression, and frailty across levels. It underscored the importance of jointly assessing these conditions and integrating within- and between-individual differences to formulate and execute targeted interventions.

## 1. Introduction

With the global population aging, psychological, and physiological health problems among middle-aged and older adults have become critical public health challenges. The proportion of individuals aged 60 years and above is projected to rise from 12% in 2015 to 22% in 2050, an increase from 900 million to 2 billion people [1]. As one of the fastest-aging countries, China had 254 million people aged 60 years and above in 2019. This number is expected to reach approximately 402 million by 2040, accounting for 28% of the national population [2, 3]. In this context, the prevalence of depressive symptoms, pain, and frailty increasingly affects the quality of life among middle-aged and older adults [4–6]. Beyond their direct physical and psychological effects, these conditions impose substantial societal and economic burdens [7–9].

Depressive symptoms are among the most common mental health disorders among middle-aged and older individuals. Globally, 280 million people are living with major depressive disorder, with a prevalence of 5.7% among adults over 60 years of age [10]. Numerous risk factors exist, with pain being a crucial one [11, 12]. The relationship between depressive symptoms and pain is bidirectional and complex, sharing pathophysiological mechanisms [13]. Evidence reveals that late-life depression exacerbates the severity and chronicity of pain [14]. A study using data from the National Comorbidity Survey found that individuals living with depression or bipolar disorder were 1.5 times more likely to develop chronic low back pain over the next 10 years than those without these conditions, and the pain progressed more rapidly [15]. According to the biopsychosocial model, affect is a more direct response to pain and is primarily regulated by the midbrain. Cognition assigns meaning to affective experiences, which in turn triggers additional emotional responses, thereby amplifying the pain experience and forming a vicious cycle of pain, psychological distress, and disability [16]. Conversely, pain can trigger or worsen depressive symptoms by limiting physical activity, disrupting social interaction, and reducing treatment effectiveness [17, 18]. Moreover, pain may exacerbate feelings of loneliness among middle-aged and older adults, further contributing to the onset of depressive symptoms [19, 20]. A review of depression and pain found that the presence of pain negatively affects the recognition and treatment of depression. Moreover, as pain progresses to moderate or severe levels, it impairs patients' functioning and becomes less responsive to treatment, which further contributes to the development and progression of depression [21].

Pain is a common and burdensome issue among middle-aged and older adults. A survey conducted across 52 countries estimated the overall age- and sex-standardized weighted prevalence of pain at 27.5%, with substantial variation across countries, ranging from 9.9% to 50.3% [22]. Pain is closely associated with frailty, as it impairs sensory, emotional, and cognitive functions, reduces daily activity levels and mobility, and thereby increases the risk of frailty [23]. Chronic pain can act as a significant stressor in the aging process, potentially initiating or accelerating the development of frailty [24]. In fact, among nonfrail older adults, chronic pain has been shown to significantly predict the onset of frailty over an average follow-up period of 5.8 years [25]. According to the disablement process model, pain, as a pathological condition, initially restricts physical activity, subsequently induces functional limitations and disability, and serves as a key precursor to the development of frailty [26]. Frailty also predicts pain. A cross-sectional study reported that frailty severity was positively associated with pain severity among older adults with cerebral palsy in a primary healthcare setting [27]. As aging progresses and frailty worsens, a chronic inflammatory state develops, contributing to both pain development and impaired joint tissue repair, often resulting in arthritis that can further intensify pain [28]. An observational study found that frailty was associated with persistent pain and higher pain intensity in hospitalized cancer patients. The impact of frailty on pain management was shown to be independent, with frailer patients more likely to experience pain that was more difficult to treat [29]. These findings underscore the strong association between pain and frailty.

Frailty is a specific syndrome characterized by multisystem impairments, decreased physical function, reduced mobility, and diminished resilience to stressors. A meta-analysis covering 62 countries reported a global frailty prevalence of 12%, with the highest prevalence observed in Africa (22%), followed by the Americas (17%), Oceania (12%), and Asia (11%) [30]. Studies have shown that frailty significantly increases the risk of developing depressive symptoms [31]. Frailty may contribute to depression through pathways such as emotional distress (e.g., feelings of worthlessness or hopelessness), reduced physical activity, and decreased social engagement [32]. When older adults are in a frail state, they often experience symptoms, such as slowed movement, fatigue, and weakness, leading them to avoid social participation. Repeated experiences of this nature may result in feelings of uselessness and emptiness, a loss of interest in previously enjoyable activities, and ultimately the development of depressive symptoms [33]. Similarly, elevated depressive symptoms can lead to weakened social relationships, reduced gait speed, and decreased physical activity, which in turn predict the onset of frailty [5]. A study on older Americans found that depression significantly increased the risk of frailty, and a meta-analysis confirmed this relationship across diverse national contexts, with findings that were consistent in many countries [34–36]. Furthermore, progression in depressive symptoms has been linked to increases in the frailty index (FI) [37]. The depression–frailty phenotype model posits that depression and frailty share common biological underpinnings, with depressive symptoms exacerbating the deterioration of both biological and behavioral pathways, thereby facilitating the development or acceleration of frailty [38].

Depression, pain, and frailty frequently co-occur in middle-aged and older adults, reinforcing one another through intertwined biological, psychological, and behavioral mechanisms [39]. Depression triggers chronic inflammation, which impairs physical function and contributes to both chronic pain and frailty [39–41]. Pain, acting as a stressor, disrupts the HPA axis, weakening stress resilience and promoting frailty, while also worsening depressive symptoms through a pain–depression feedback loop [41]. Frailty, in turn, reduces physical capacity, increases fatigue and dependance, further intensifying both depression and pain [42, 43]. Together, these conditions form a pathological cycle driven by inflammation, physical decline, emotional distress, and behavioral dysfunction, with their reciprocal and cross-lagged interactions compounding adverse outcomes.

Despite increasing recognition of these interconnections, research into the dynamic interplay between depressive symptoms, pain, and frailty remains limited. Existing studies support the view that these conditions are interrelated both bidirectionally and multidimensionally [44–46]. Depressive symptoms increase pain sensitivity and accelerate the onset of frailty, while pain and frailty in turn worsen depressive symptoms. However, most previous studies have relied on cross-sectional data, which cannot establish temporal sequencing and thus fail to clarify causal relationships [47–49]. To bridge this gap, longitudinal research is needed to better understand the temporal and reciprocal associations among depressive symptoms, pain, and frailty.

Cross-lagged panel modeling (CLPM) offers a promising approach for investigating these relationships. It allows researchers to examine how changes in one variable predict future changes in another, thereby shedding light on potential causal pathways. However, traditional CLPM does not distinguish between stable individual traits and state-level fluctuations, potentially biasing the results. The random-intercept CLPM (RI-CLPM) addresses this limitation by incorporating a random intercept, effectively separating trait-like characteristics from time-dependent factors and improving the accuracy of parameter estimation.

This study uses nationally representative longitudinal data on middle-aged and older adults in China. Both CLPM and RI-CLPM are employed to investigate the temporal relationships among depressive symptoms, pain, and frailty. This study aims to explore the reciprocal associations among these depressive symptoms, pain, and frailty among middle-aged and older adults, offer novel insights into aging-associated health challenges, and inform effective prevention and intervention strategies.

## 2. Methods

### 2.1. Study Design

**Figure 1:**
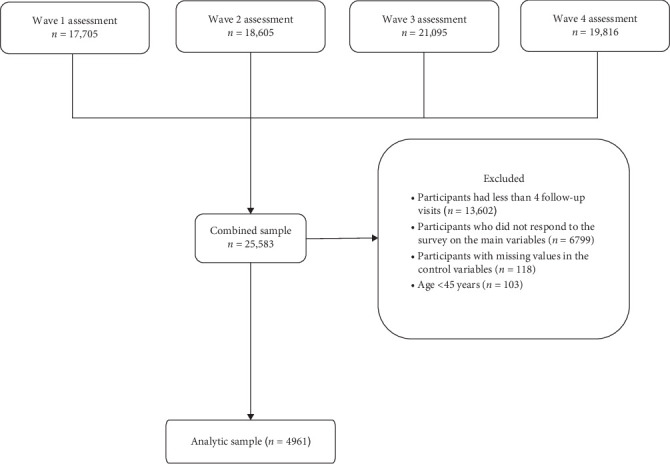
Flowchart of study participants.

This study used data from the China Health and Retirement Longitudinal Study (CHARLS). CHARLS is an ongoing, nationally representative longitudinal survey targeting Chinese adults aged 45 and above [50]. The baseline survey, conducted in 2011, used a multistage stratified probability sampling method proportional to population size, recruiting 17,708 participants from 28 provinces across the country. All participants were followed up every two to 3 years through face-to-face, computer-assisted personal interviews. The first three surveys included physical measurements, with gait speed assessments limited to participants aged 60 and above. The study protocol was approved by the Peking University Institutional Review Board (IRB00001052-11015), and all participants provided written informed consent before the survey. Information about the survey design and data collection of CHARLS can be obtained from the official website http://charls.pku.edu.cn/. Data were collected from four survey waves between 2011 and 2018. The present study included individuals who completed the four follow-up interviews and reported depressive symptoms, pain, and frailty. After excluding participants with missing data on key variables and ineligible respondents, the final sample comprised 4961 middle-aged and older adults aged 45 and above. The specific process of sample selection is shown in Figure 1.

## 3. Measurements

### 3.1. Depressive Symptom

Depressive symptoms were assessed using the 10-item Center for Epidemiological Studies Depression Scale (CESD-10), which has been extensively validated for use in general populations [51]. The CESD-10 demonstrates sufficient reliability and validity when used among the community-dwelling older adults in China [52]. It contains 10 items assessing participants' feelings and behaviors over the past week, with eight items measuring the frequency of various depression-related symptoms, and two questions assessing the frequency of positive emotions in the past week. Each item offers four response options: “rarely or none of the time” (0), “some or a little of the time” (1), “occasionally or a moderate amount of time” (2), and “most or all of the time” (3). Positive emotion items were reverse-scored. The total score ranges from 0 to 30, with higher scores indicating greater depressive severity.

### 3.2. Pain

Pain was assessed using two self-reported items: “Are you often troubled with body pains?” and “On what part of your body do you feel pain? Please list all parts of the body where you are currently feeling pain.” Participants who did not report any pain were assigned a score of zero. Those who reported experiencing pain were asked to indicate the affected areas on a validated anatomical chart, which included body regions, such as the head, shoulders, and arms. Each reported pain site was assigned one score, resulting in a total pain score (PA) ranging from 0 to 15, reflecting the extent of pain across multiple body regions [53].

### 3.3. Frailty

Frailty was assessed by the FI, which is constructed based on the accumulation of various health deficits associated with aging. In this study, FI was meticulously developed in standardized procedures. Previous research has demonstrated that an index containing 30–40 variables is sufficient for accurately predicting adverse health outcomes [54]. Therefore, this study selected 36 indicators to construct FI, encompassing aspects, such as disease, disability, activities of daily living (ADL), and mobility. Each health variable was dichotomized (0 indicating absence of a health deficit; 1 signifying its presence). FI was calculated as the ratio of the total number of existing health deficits to the total number of potential deficits (36), yielding a score ranging from 0 to 1, with higher scores indicating greater frailty. The specific items used to construct the FI are detailed in Supporting Information 1: Table S1.

### 3.4. Control Variables

Along with previous studies [55–57], the present study included sociodemographic characteristics, lifestyle factors, and major clinical factors as potential covariates. Sociodemographic characteristics included age, sex, marital status (married or other), residence (urban or rural), and educational attainment (no formal education, primary school, middle school, or high school and above). Lifestyle factors included smoking status (categorized as current nonsmoker and smoker; individuals who never smoked or had quit smoking were classified as current nonsmokers), drinking status (classified as nondrinker if no alcohol was consumed in the past year, and drinker otherwise), length of night sleep, and length of noon sleep. For major clinical factors, self-perceived health status was assessed. Age, sex, and education were measured only at baseline (time-independent covariates), while other variables (time-dependent covariates) were measured across all four surveys.

### 3.5. Statistical Analysis

To ensure data reliability, missing values in the frailty variable related to ADL assessment were imputed using the k-nearest neighbor (KNN) algorithm, which has demonstrated effectiveness and feasibility for handling similar datasets [58]. This study employed CLPM and RI-CLPM to investigate the relationships between depressive symptoms, pain, and frailty at both interindividual and within-individual levels. CLPM assumes that variables influence each other over time and that individuals start from similar baseline conditions, primarily capturing between-person differences rather than individual-specific changes [59]. In contrast, RI-CLPM extends CLPM by incorporating random intercepts, which control for stable individual differences and allow researchers to infer within-person relationships among variables [60]. This model accounts for baseline differences between individuals, enabling analyses to focus on temporal changes within the same individual and emphasizing within-individual effects.

First, descriptive statistical analyses were conducted to describe the characteristics of the study participants. For continuous variables that follow a normal distribution, means and standard deviations were reported; otherwise, medians and interquartile ranges were used; and for categorical variables, frequencies and percentages were reported.

Second, Spearman correlation analyses were performed to examine the relationships among depressive symptoms, pain, and frailty both within and across time points. Subsequently, CLPM and RI-CLPM were used to explore the bidirectional relationships among these variables. To control for potential effects of covariates on effect sizes and to improve model fitness, we constructed six sequential models. Model 1 utilizes the CLPM to examine the interpersonal effects of depressive symptoms, pain, and frailty. This model included autoregressive paths, cross-lagged effects, and contemporaneous correlations. This model serves to preliminarily test the temporal sequence and mutual influence among the three variables and observe their basic dynamic connections. Model 2 extends Model 1 by incorporating three time-independent covariates: age, gender, and education. These are key demographic factors affecting middle-aged and elderly health, used to control stable individual differences for a more accurate assessment of cross-lagged paths between variables. Building further on Model 2, Model 3 includes additional time-dependent covariates, namely marital status, residence, smoking status, drinking status, length of night sleep, length of noon sleep, and self-perceived health status. These variables reflect the dynamic changes in individuals' lifestyles and social environments over time, helping to better control for within-individual temporal confounding factors. This approach enables us to assess the stability and independence of observed effects in increasingly complex models, consistent with recommendations from prior cross-lagged modeling studies [61]. Model 4 employed RI-CLPM to examine within-person relationships while controlling for stable individual traits. Model 5, based on Model 4, additionally included three time-invariant covariates: age, gender, and educational level. Model 6 further built upon Model 5 by incorporating several time-varying covariates, specifically marital status, place of residence, smoking status, drinking status, length of night sleep, length of noon sleep, and self-perceived health status. All models were estimated using robust maximum likelihood estimation (MLR), which accommodates nonnormal and nonindependent data. Model fit was assessed using several indices: the comparative fit index (CFI), Tucker–Lewis index (TLI), root mean square error of approximation (RMSEA), and standardized root mean square residual (SRMR). Good model fit was defined by CFI > 0.90, TLI > 0.90, RMSEA < 0.08, and SRMR < 0.08. Additionally, lower Akaike information criterion (AIC) and Bayesian information criterion (BIC) values indicated better model performance. It should be noted that previous research has demonstrated that some fit indices in CLPM models may be acceptably below thresholds [62]. To test the robustness of the research findings, we conducted two sensitivity analyses. First, a cross-lagged analysis was performed on the population aged 60 and above. Second, the analyses were replicated, but excluding participants with high school and above. All statistical analyses were conducted using RStudio 4.4.0 and Mplus 8.3, and statistical significance was set at *p* < 0.05.

## 4. Results

### 4.1. Characteristics of Study Participants

Table 1 summarizes the baseline characteristics of the participants. It can be observed that 2514 participants (50.7%) were male. The majority of the participants (68.4%) were aged between 45 and 60 years. Most participants (64.1%) perceived their general health status as average (49.7%). In terms of functional status, 51.8% of the participants reported limitations in ADL, while the vast majority (85.8%) indicated no limitations in instrumental ADL (IADL). Most participants resided in rural areas (84.1%) and were married (88.3%). A substantial portion of the participants (69.3%) had one or more chronic diseases. Regarding sleep patterns, 42.7% of the participants slept 6–8 h at night, and 63.9% of them took naps of less than 60 min.

### 4.2. Correlation Between Depressive Symptoms, Pain, and Frailty

Spearman correlation coefficients for depressive symptoms, pain, and frailty across four time points (2011, 2013, 2015, and 2018). indicate that each variable exhibited significant positive correlations across time, with depressive symptoms demonstrating the highest temporal stability (Table 2). Regarding the associations between variables, pain showed a moderate positive correlation with depressive symptoms across all time points. The correlation between baseline frailty and subsequent depressive symptoms weakened over time, suggesting that these constructs may capture distinct underlying traits.

### 4.3. Model Test of the Relations Between Depressive Symptoms, Pain, and Frailty

Table 3 summarizes the fit indices for the six models. Results indicate that Model 1 demonstrated relatively poor fit (CFI = 0.858, TLI = 0.668, RMSEA = 0.104, SRMR = 0.076). Model 2 (CFI = 0.896, TLI = 0.608, RMSEA = 0.100, SRMR = 0.060) showed improvement. Model 3 exhibited further enhancement in fit (CFI = 0.892, TLI = 0.831, RMSEA = 0.038, SRMR = 0.041), suggesting that the inclusion of covariates accounted for substantial variability in the primary variables, thereby allowing for more precise estimates of their relationships [63]. Consequently, Model 3 was selected to examine the between-person effects of depressive symptoms, pain, and frailty.

Model 4 demonstrated good fit (CFI = 0.992, TLI = 0.974, RMSEA = 0.029, SRMR = 0.018), meeting all recommended critical values. This indicates that the addition of the random intercept significantly improves model performance. Both Model 5 (CFI = 0.985, TLI = 0.967, RMSEA = 0.029, SRMR = 0.020) and Model 6 (CFI = 0.942, TLI = 0.915, RMSEA = 0.027, SRMR = 0.045) exhibited good fit. All fit indices for Model 6 met the required critical values. Furthermore, Model 6 incorporated covariates and demonstrated relatively low AIC and BIC values. Therefore, Model 6 was selected to examine the within-individual effects of depressive symptoms, pain, and frailty.

### 4.4. Relationship Among Depressive Symptoms, Pain, and Frailty

**Figure 2:**
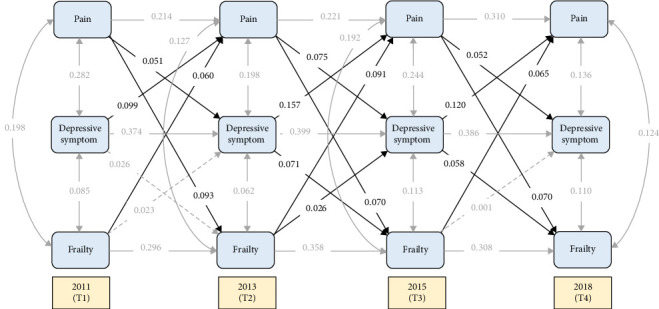
Cross-lagged panel model (CLPM). The solid lines represent significance, while the dashed lines represent nonsignificance. For the sake of brevity, all covariates were estimated in the analysis but not shown in the diagram.

Supporting Information 2: Table S2 presents the standardized coefficients for autoregressive paths, cross-lagged paths, and synchronous correlations among PAs, depression symptom scores (DSs), and FI across six models examining their relationships in middle-aged and older Chinese adults. Pain significantly predicted depressive symptoms at all time points (*β* = 0.051, *p*=0.002; *β* = 0.075, *p* < 0.001; *β* = 0.052, *p*=0.001) (Figure 2). Depressive symptoms also demonstrated significant predictive effects on pain (*β*s = 0.099–0.157, *p*s < 0.001). Similarly, pain showed significant positive predictive effects on frailty (*β*s = 0.070–0.093, *p*s < 0.001), and frailty significantly predicted subsequent pain (*β*s = 0.065–0.091, *p*s < 0.001). In contrast, the temporal relationship between frailty and depressive symptoms was relatively weaker and exhibited a declining trend. During the period from T2 to T3, frailty positively predicted depressive symptoms (*β* = 0.026, *p*=0.041), and depressive symptoms also predicted frailty (β = 0.071, *p* < 0.001). However, in the period from T3 to T4, only depressive symptoms unidirectionally predicted frailty (*β* = 0.058, *p*=0.001).

**Figure 3:**
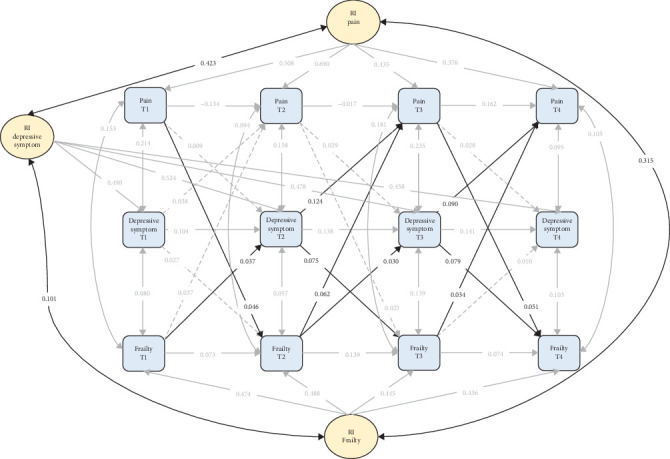
Random intercept cross-lagged panel models (RI-CLPMs). The solid lines represent significance, while the dashed lines represent nonsignificance. For the sake of brevity, all covariates were estimated in the analysis but not shown in the diagram.

After accounting for individual heterogeneity, the RI-CLPM revealed a distinctly different pattern of relationships. When controlling for between-person differences, many previously significant cross-lagged effects diminished or became nonsignificant (Figure 3). Pain no longer significantly predicted depressive symptoms across any of the time points. The predictive effect of depressive symptoms on pain, while still present, became relatively weaker for the T2 to T3 period (*β* = 0.124, *p* < 0.001) and the T3 to T4 period (*β* = 0.090, *p* < 0.001). Similarly, the relationship between pain and frailty showed an attenuated trend. Specifically, in the T1 to T2 period, only pain significantly predicted frailty (*β* = 0.046, *p*=0.017). In the T2 to T3 period, frailty unidirectionally predicted pain (*β* = 0.062, *p* < 0.001). During the T3 to T4 period, pain and frailty demonstrated significant bidirectional prediction, with pain positively predicting frailty (*β* = 0.051, *p*=0.006), and frailty predicting pain (*β* = 0.034, *p*=0.027). In contrast to the attenuated relationships described above, the reciprocal relationship between frailty and depressive symptoms subtly intensified in the RI-CLPM. From T1 to T2, frailty positively predicted depressive symptoms (*β* = 0.037, *p*=0.013), a relationship that persisted from T2 to T3 (*β* = 0.030, *p*=0.034). The predicting effect of depressive symptoms on frailty showed a similar consistent pattern: T2 to T3 (*β* = 0.075, *p* < 0.001); T3 to T4 (*β* = 0.079, *p* < 0.001), indicating an overall strengthening trend. Furthermore, the RI-CLPM identified significant between-person correlations among the random intercepts, with the strongest latent trait correlation observed between pain and depressive symptom (*β* = 0.423, *p* < 0.001), followed by pain and frailty (*β* = 0.315, *p* < 0.001). In contrast, the correlation between depressive symptoms and frailty was relatively weaker (*β* = 0.101, *p*=0.037). These findings underscore the complex interrelationships among these health issues and highlight the crucial role of individual differences in shaping these associations.

### 4.5. Sensitivity Analysis

First, we conducted sensitivity analyses by restricting the sample to individuals aged 60 and older (*n* = 1783). Results from CLPM were substantially consistent with the primary analysis results. However, the RI-CLPM yielded slightly different findings. Specifically, the predictive effect of pain on frailty was no longer significant, as did the association between frailty and depressive symptoms (Supporting Information 3: Table S3). A possible explanation for these differences is that older adults, having accumulated rich aging-related experience, may be better equipped to manage adverse health issues such as frailty[64]. Additionally, with increased awareness of the finite nature of time, older adults may actively regulate emotions, enhancing positive emotions, while reducing negative emotions[65]. These age-related compensatory mechanisms may attenuate the relationships between pain, frailty, and depressive symptoms in the oldest segment of our sample. Second, to assess the robustness of our findings across educational backgrounds, we performed an additional analysis excluding participants with high school and above (*n* = 713; remaining *n* = 4248). This analysis also confirmed the stability of our findings (Supporting Information 4: Table S4). Finally, we restricted the population to married individuals (*n* = 4381) and conducted an analysis, which yielded results consistent with the primary analysis (Supporting Information 5: Table S5).

## 5. Discussion

This study analyzed 7 years of longitudinal data from four waves of the CHARLS, employing CLPM and RI-CLPM to investigate the dynamic associations among depressive symptoms, pain, and frailty in middle-aged and older adults. The CLPM results indicated that pain predicted subsequent worsening in both depressive symptoms and frailty, while depressive symptoms also contributed to greater pain and frailty over time. Frailty also has a significant lagged effect on pain. However, the relationship between frailty and depression appeared less stable. By accounting for individual heterogeneity, the RI-CLPM revealed more nuanced within-person dynamics. After controlling for stable trait differences, the effect of pain on depressive symptoms was no longer significant, and the predictive effect between pain and frailty was weakened.

The complex interrelationships identified in this study can be understood through several theoretical frameworks. Pain worsens depressive symptoms as it often restricts daily activities and social participation, leading to functional impairment and loss of social roles, both of which contribute to depressive symptoms [18]. The fear–avoidance model further explains this link. Fear of pain prompts avoidance behaviors, which in turn reinforce pain-related distress and increase the risk of developing depression [66, 67]. Inflammation also plays an important role in mediating the relationship between pain and depressive symptoms. Individuals experiencing pain tend to exhibit elevated levels of inflammatory markers, which are associated with depressive symptomatology [68, 69]. Furthermore, persistent pain activates the neuroendocrine system and promotes the release of pro-inflammatory cytokines, accelerating the onset and progression of frailty [70]. This is consistent with the “inflamm-aging” theory, which posits that chronic inflammation underlies many age-related health conditions [71, 72]. Pain-related activity limitations often result in sedentary behavior, further increasing inflammatory responses and contributing to frailty development [73]. A prolonged imbalance between anti- and pro-inflammatory processes exacerbates both frailty and depressive outcomes [74]. Among middle-aged and older adults, pain is linked to declines in gait speed, grip strength, and nutrition status, all of which are key indicators of frailty [75, 76]. Moreover, co-occurring issues, such as sleep disturbances and malnutrition, reduce physiological reserves, heightening the risk of frailty, falls, and disability, supporting the “pain homeostenosis” hypothesis [77].

The impact of depressive symptoms on pain and frailty is multifaceted. Depressive symptoms can reduce physical activity, impair sleep quality, and elevate levels of pro-inflammatory cytokines, all of which contribute to the worsening of both pain and frailty [78–80]. Moreover, depressive symptom are associated with increased risk of fractures, bone loss, and muscle atrophy, leading to significant mobility limitations and even disability, further accelerating the development of frailty [81, 82]. Depressive symptoms also negatively affect treatment adherence and health behaviors, indirectly influencing and hindering effective pain management and contributing to frailty progression [83, 84]. The cognitive vulnerability model holds that adverse life events can lead to the development of negative cognitive schemas, resulting in pessimistic attitudes and self-perceptions [85]. These cognitive distortions affect how individuals perceive and evaluate pain and physical conditions, intensifying their experience of suffering. Furthermore, stigma mediates the relationship between depressive symptoms and pain. Depression is often more heavily stigmatized than pain in the Chinese context, which may lead individuals to conceal their pain symptoms rather than depressive symptoms. This underreporting can delay appropriate treatment and result in the worsening of pain over time [86]. Interestingly, the lagged effect of early depression on frailty was not significant in this study. This may be attributed to timely medical interventions, as well as social support systems, which may buffer the progression of frailty in the early stages of depression. However, as depressive symptoms persist and treatment resistance increases, the relationship between depressive symptoms and frailty becomes more pronounced [87, 88]. Notably, a recent bidirectional Mendelian randomization study found no evidence that psychiatric disorders, including major depressive disorder, are causal risk factors when analyzed in reverse. This discrepancy may reflect differences in demographic profiles, study designs, or diagnostic criteria [89].

Frailty is a geriatric syndrome characterized by the progressive decline of physiological systems, rendering individuals more vulnerable to stressors and increasing their risk of adverse health outcomes [90]. The activity limitations associated with frailty can lead to social isolation and emotional distress, partially explaining its influence on the onset of depressive symptoms [91, 92]. Inflammatory pathways also underpin the correlation between frailty and depressive symptoms, with biomarkers such as C-reactive protein and leukocytes acting as essential mediators [93]. Chronic diseases of systems such as the urinary and digestive systems in frail patients can lead to the occurrence of pain and, to a large extent, promote the development of pain [94, 95]. A longitudinal study across multiple countries found that individuals in the prefrail state or frail state have a significantly higher prevalence and degree of pain when compared with those in a robust state. This study also reported that pain in areas such as the head, neck, trunk, and extremities is strongly associated with frailty [96]. Moreover, frailty-related sensory impairments, such as visual and hearing loss, can increase pain sensitivity and hinder effective pain communication [97, 98]. This can delay diagnosis and treatment, further exacerbating the pain experience. People with visual and hearing impairments may internalize stigma, which can hinder social interaction and increase their risk of isolation. They also have a higher likelihood of experiencing many traumatic events, such as bullying, leading to social withdrawal and thus depressive symptoms [99, 100]. However, despite these associations, the impact of frailty on depressive symptoms appears to be inconsistent. An 8-year trajectory study found that while frailty and cognitive function steadily declined over time, depressive symptoms remained relatively stable [101]. Some individuals in a prefrail or frail state may experience short-term recovery, but these improvements tend to have minimal influence on depressive symptoms, which are more strongly linked to significant negative health transitions [102]. Overall, the relationship between depressive symptoms and frailty is complex and warrants further investigation to clarify the underlying mechanisms.

Notably, the RI-CLPM results revealed that, after accounting for individual differences, the lagged effect of pain on depressive symptoms was no longer significant, and the association between pain and frailty was substantially attenuated. These findings contrast with earlier studies that reported stronger predictive relationships among these variables [44, 46]. This divergence suggests that individual characteristics, such as age, sex, and educational attainment, may shape the developmental trajectories of depressive symptoms, pain, and frailty [103]. For example, individuals who are older, female, less educated, or who report poor self-perceived health status are more likely to experience pain and report more severe depressive symptoms [104]. In addition, some important traits should not be overlooked. While pain may trigger emotional deterioration or emotional blunting in some individuals, others may benefit from increased social and emotional support in response to pain [67]. A meta-analysis found no association between pain and depressive symptoms in cases of acute pain; however, among individuals with chronic pain, depressive symptoms were significantly associated with higher pain intensity, disability, and poorer recovery [105]. These findings underscore the importance of pain duration and intensity in shaping its psychological consequences. It is worth noting that other studies have shown that lower levels of pain may paradoxically be associated with a greater risk of developing major depressive disorder. For example, in people with hip fractures, those reporting no pain (pain level 1) had a higher risk of developing depression compared to those with mild pain (pain level 2), possibly due to attentional shifts toward physical discomfort that temporarily suppress emotional distress [106]. Pain tolerance, which varies across individuals, may also mediate the relationship between pain and depressive symptoms. The anatomical site of pain further moderates this relationship. In people living with lower back pain, those whose symptoms radiate below the knee tend to report poorer quality of life compared to those with localized or proximal pain, which, in turn, increases the risk of depression [107, 108]. Furthermore, researchers found that while depression is associated with pain across multiple body regions, prevalence varies considerably depending on location, with chest and abdominal pain showing the strongest associations, and lower back pain the weakest [109]. We can also observe that frailty continues to predict subsequent depression. This indicates that the dynamic increase in an individual's frailty level can significantly predict the subsequent fluctuations in their depressive symptoms, and this result is consistent with previous research [110]. This within-individual association may reflect the psychological burden and the decline in functional autonomy experienced during periods of increased frailty, as well as common biological mechanisms, such as chronic inflammation and neuroendocrine disorders [32, 38, 111, 112]. Collectively, these findings highlight the necessity of accounting for both interindividual and within-individual variability when analyzing the complex interplay among depressive symptoms, pain, and frailty.

This study identifies complex and interrelated relationships among depressive symptoms, pain, and frailty in middle-aged and older Chinese adults. The findings suggest that these conditions may mutually reinforce one another, creating a detrimental cycle that exacerbates health outcomes over time. Early identification and timely, targeted interventions are therefore essential to disrupt this cycle. Clinical management should consider both the intensity and location of pain, as these factors may significantly influence psychological and physical trajectories. Future research should continue to explore the underlying biological, psychological, and social mechanisms driving these associations. In light of our findings, we advocate for the routine assessments of pain, mental health, and physical functioning in aging populations, particularly among individuals identified as high-risk based on demographic or health-related characteristics. To effectively reduce the long-term burden of these interconnected conditions, policymakers should prioritize strengthening social support systems and enhancing healthcare accessibility.

## 6. Strengths and Limitations

This research offers several strengths. First, it draws on four-wave, 7-year nationally representative data from the CHARLS, ensuring high statistical power and generalizability to China's middle-aged and older adult population. Second, by simultaneously examining the interrelationships among depressive symptoms, pain, and frailty, this study presents a comprehensive understanding of their dynamic interactions, offering a theoretical foundation for integrated intervention strategies tailored to the needs of the aging population in China. Third, the application of both the CLPM and RI-CLPM allows for the differentiation between within-individual and between-individual variations, thus improving the accuracy and interpretability of the results. Finally, the inclusion of key covariates, such as demographic and lifestyle factors, improves the robustness and clinical relevance of the findings.

However, this study is subject to several limitations. Although self-reported data on pain, depressive symptoms, and frailty have been validated within the Chinese context, reliance on subjective data may introduce reporting bias. Additionally, attrition over the follow-up period may limit the representativeness of the study sample, potentially skewing results toward healthier individuals. The 7-year follow-up duration, coupled with the relatively long intervals between survey waves, may also limit the ability to detect short-term fluctuations and transitions in these conditions. Lastly, the conservative specification of our models may constrain the detection of more complex or nonlinear relationships. Future research should consider incorporating objective assessments, biomarkers, and more frequent data collection to gain insights into the mechanisms underpinning these interrelated health issues.

## 7. Conclusions and Implications

In conclusion, this study identified cross-lagged associations among depressive symptoms, pain, and frailty at both the interindividual and within-individual levels through analyzing 7 years of longitudinal data from CHARLS. The CLPM analysis indicated significant bidirectional relationships between pain and depressive symptoms, as well as between pain and frailty. However, when individual differences were accounted for using RI-CLPM, the effect of pain on depressive symptoms was no longer statistically significant. These findings highlight the necessity of distinguishing between within-individual differences and between-individual dynamics in understanding the development and interplay of these conditions. They also highlight the critical need for early identification and timely intervention to prevent the compounding effects of these conditions. Future research should aim to further elucidate the underlying biological and psychological mechanisms that drive these associations.

## Figures and Tables

**Table 1:** Participant characteristics reported or measured at baseline (*n* = 4961).

Variable	*n* (%)
Age (years)	
45–60	3393 (68.4)
61–75	1497 (30.2)
>75	71 (1.4)
Gender	
Female	2447 (49.3)
Male	2514 (50.7)
Education level	
High school and above	713 (14.4)
Middle school	1260 (25.4)
Primary school	2024 (40.8)
Illiterate	964 (19.4)
Smoking	
No	3330 (67.1)
Yes	1631 (32.9)
Drinking	
No	3199 (64.5)
Yes	1762 (35.5)
Self-perceived health status	
Good	1394 (28.1)
Fair	2468 (49.7)
Poor	1099 (22.2)
ADL	
No	2390 (48.2)
Yes	2571 (51.8)
IADL	
No	4258 (85.8)
Yes	703 (14.2)
Residence status	
City	790 (15.9)
Rural	4171 (84.1)
Marital status	
Married	4381 (88.3)
Others	580 (11.7)
Chronic disease status	
No	1791 (36.1)
Yes	3170 (63.9)
Length of night sleep (h)	
<6	1291 (26.0)
6–8	2117 (42.7)
≥8	1553 (31.3)
Length of noon sleep (min)	
<60	3170 (63.9)
60–120	1267 (25.5)
≥120	524 (10.6)

Abbreviations: ADL, activities of daily living; IADL, instrumental activity of daily living.

**Table 2:** Spearman correlations between depressive symptoms, pain, and frailty at T1, T2, T3, and T4.

Variables	Median (IQR)	FI_T1	FI_T2	FI_T3	FI_T4	PA_T1	PA_T2	PA_T3	PA_T4	DS_T1	DS_T2	DS_T3	DS_T4
FI_T1	0.167 (0.083, 0.194)	1	—	—	—	—	—	—	—	—	—	—	—
FI_T2	0.167 (0.083, 0.194)	0.291*⁣*^*∗∗∗*^	1	—	—	—	—	—	—	—	—	—	—
FI_T3	0.167 (0.083, 0.194)	0.281*⁣*^*∗∗∗*^	0.344*⁣*^*∗∗∗*^	1	—	—	—	—	—	—	—	—	—
FI_T4	0.139 (0.056, 0.194)	0.168*⁣*^*∗∗∗*^	0.234*⁣*^*∗∗∗*^	0.286*⁣*^*∗∗∗*^	1	—	—	—	—	—	—	—	—
PA_T1	0.000 (0.000, 1.000)	0.089*⁣*^*∗∗∗*^	0.093*⁣*^*∗∗∗*^	0.107*⁣*^*∗∗∗*^	0.106*⁣*^*∗∗∗*^	1	—	—	—	—	—	—	—
PA_T2	0.000 (0.000, 1.000)	0.071*⁣*^*∗∗∗*^	0.079*⁣*^*∗∗∗*^	0.129*⁣*^*∗∗∗*^	0.118*⁣*^*∗∗∗*^	0.315*⁣*^*∗∗∗*^	1	—	—	—	—	—	—
PA_T3	0.000 (0.000, 0.000)	0.063*⁣*^*∗∗∗*^	0.101*⁣*^*∗∗∗*^	0.178*⁣*^*∗∗∗*^	0.141*⁣*^*∗∗∗*^	0.356*⁣*^*∗∗∗*^	0.359*⁣*^*∗∗∗*^	1	—	—	—	—	—
PA_T4	1.000 (0.000, 4.000)	0.047*⁣*^*∗∗∗*^	0.049*⁣*^*∗∗∗*^	0.125*⁣*^*∗∗∗*^	0.133*⁣*^*∗∗∗*^	0.325*⁣*^*∗∗∗*^	0.356*⁣*^*∗∗∗*^	0.407*⁣*^*∗∗∗*^	1	—	—	—	—
DS_T1	6.000 (3.000, 11.000)	0.028*⁣*^*∗*^	0.045*⁣*^*∗∗*^	0.071*⁣*^*∗∗∗*^	0.106*⁣*^*∗∗∗*^	0.403*⁣*^*∗∗∗*^	0.270*⁣*^*∗∗∗*^	0.321*⁣*^*∗∗∗*^	0.325*⁣*^*∗∗∗*^	1	—	—	—
DS_T2	6.000 (3.000, 10.000)	0.037*⁣*^*∗∗*^	0.039*⁣*^*∗∗*^	0.100*⁣*^*∗∗∗*^	0.114*⁣*^*∗∗∗*^	0.275*⁣*^*∗∗∗*^	0.346*⁣*^*∗∗∗*^	0.334*⁣*^*∗∗∗*^	0.336*⁣*^*∗∗∗*^	0.478*⁣*^*∗∗∗*^	1	—	—
DS_T3	6.000 (3.000, 10.000)	0.020	0.044*⁣*^*∗∗*^	0.115*⁣*^*∗∗∗*^	0.112*⁣*^*∗∗∗*^	0.292*⁣*^*∗∗∗*^	0.287*⁣*^*∗∗∗*^	0.420*⁣*^*∗∗∗*^	0.342*⁣*^*∗∗∗*^	0.461*⁣*^*∗∗∗*^	0.510*⁣*^*∗∗∗*^	1	—
DS_T4	7.000 (3.000, 12.000)	0.010	0.038*⁣*^*∗∗*^	0.067*⁣*^*∗∗∗*^	0.126*⁣*^*∗∗∗*^	0.260*⁣*^*∗∗∗*^	0.261*⁣*^*∗∗∗*^	0.316*⁣*^*∗∗∗*^	0.363*⁣*^*∗∗∗*^	0.416*⁣*^*∗∗∗*^	0.478*⁣*^*∗∗∗*^	0.504*⁣*^*∗∗∗*^	1

Abbreviations: DS, depressive symptoms; FI, frailty index; IQR, interquartile range; PA, pain.

*⁣* ^*∗*^ *p* < 0.05.

*⁣* ^*∗∗*^ *p* < 0.01.

*⁣* ^*∗∗∗*^ *p* < 0.001.

**Table 3:** Model fit indices of the six models.

Model	*χ* ^2^	df	CFI	TLI	RMSEA	SRMR	AIC	BIC
Model 1	1481.117	27	0.858	0.668	0.104	0.076	168,177.905	168,587.995
Model 2	1371.305	27	0.896	0.608	0.100	0.060	167,346.640	167,991.067
Model 3	2281.919	279	0.892	0.831	0.038	0.041	164,318.550	165,509.763
Model 4	111.095	21	0.992	0.974	0.029	0.018	166,335.489	166,784.635
Model 5	247.227	48	0.985	0.967	0.029	0.020	165,731.639	166,239.370
Model 6	1380.935	300	0.942	0.915	0.027	0.045	163,353.263	164,407.780

*Note:* Model 1 = Cross-lagged panel model (CLPM). Model 2 = Cross-lagged panel model (CLPM) adjusting for gender, age, and educational level. Model 3 = Cross-lagged panel model (CLPM) adjusting for gender, age, educational level, length of night sleep, length of noon sleep, smoking, drinking, self-perceived health status, residence, and marital status. Model 4 = Random intercept cross-lagged panel model (RI-CLPM). Model 5 = Random intercept cross-lagged panel model (RI-CLPM), adjusting for variables the same as Model 2. Model 6 = Random intercept cross-lagged panel model (RI-CLPM), adjusting for variables the same as Model 3.

## Data Availability

The data used in this study are publicly available on the CHARLS website: https://charls.pku.edu.cn.

## Supporting Information

Supporting Information 1 Table S1. Details the specific items of the frailty index. The 36 items used to construct the frailty index.

Supporting Information 2 Table S2. Lists of the standardized coefficients of the main analysis. Standardization coefficient results of depressive symptoms, pain, and frailty.

Supporting Information 3 Table S3. Standardization coefficient results of depressive symptoms, pain, and frailty.

Supporting Information 4 Table S4. Standardization coefficient results of depressive symptoms, pain, and frailty, present the results of two sensitivity analyses.

Supporting Information 5 Table S5. Standardization coefficient results of depressive symptoms, pain, and frailty.

## References

[1] Cui J., Shibata Y., Zhu T., Zhou J., Zhang J. (2022). Osteocytes in Bone Aging: Advances, Challenges, and Future Perspectives. *Ageing Research Reviews*.

[2] Jiang Q., Feng Q. (2022). Editorial: Aging and Health in China. *Frontiers in Public Health*.

[3] The Lancet (2022). Population Ageing in China: Crisis or Opportunity?. *The Lancet*.

[4] D’Agnelli S., Amodeo G., Franchi S. (2022). Frailty and Pain, Human Studies and Animal Models. *Ageing Research Reviews*.

[5] Soysal P., Veronese N., Thompson T. (2017). Relationship Between Depression and Frailty in Older Adults: A Systematic Review and Meta-Analysis. *Ageing Research Reviews*.

[6] Zis P., Daskalaki A., Bountouni I., Sykioti P., Varrassi G., Paladini A. (2017). Depression and Chronic Pain in the Elderly: Links and Management Challenges. *Clinical Interventions in Aging*.

[7] Cohen S. P., Vase L., Hooten W. M. (2021). Chronic Pain: An Update on Burden, Best Practices, and New Advances. *Lancet*.

[8] Hoogendijk E. O., Afilalo J., Ensrud K. E., Kowal P., Onder G., Fried L. P. (2019). Frailty: Implications for Clinical Practice and Public Health. *The Lancet*.

[9] McCarron R. M., Shapiro B., Rawles J., Luo J. (2021). Depression. *Annals of Internal Medicine*.

[10] World Health Organization (2020). Depression. https://www.who.int/news-room/fact-sheets/detail/depression.

[11] O’Connor S. J., Hewitt N., Kuc J., Orsini L. S. (2023). Predictors and Risk Factors of Treatment-Resistant Depression: A Systematic Review. *The Journal of Clinical Psychiatry*.

[12] Soltani S., Noel M., Bernier E., Kopala-Sibley D. C. (2023). Pain and Insomnia as Risk Factors for First Lifetime Onsets of Anxiety, Depression, and Suicidality in Adolescence. *Pain*.

[13] Tappe-Theodor A., Kuner R. (2019). A Common Ground for Pain and Depression. *Nature Neuroscience*.

[14] Ryan S., McGuire B. (2016). Psychological Predictors of Pain Severity, Pain Interference, Depression, and Anxiety in Rheumatoid Arthritis Patients With Chronic Pain. *British Journal of Health Psychology*.

[15] Schmaling K. B., Nounou Z. A. (2019). Incident Chronic Spinal Pain and Depressive Disorders: Data From the National Comorbidity Survey. *The Journal of Pain*.

[16] Gatchel R. J., Peng Y. B., Peters M. L., Fuchs P. N., Turk D. C. (2007). The Biopsychosocial Approach to Chronic Pain: Scientific Advances and Future Directions. *Psychological Bulletin*.

[17] Martini M., Arenhardt F. K., Caldieraro M. A. (2023). Chronic Pain Predicts a Worse Response to Depression Treatment, Regardless of Thyroid Function or Psychotropics Prescribed. *Journal of Affective Disorders*.

[18] Yong R. J., Mullins P. M., Bhattacharyya N. (2022). Prevalence of Chronic Pain Among Adults in the United States. *Pain*.

[19] Loeffler A., Steptoe A. (2021). Bidirectional Longitudinal Associations Between Loneliness and Pain, and the Role of Inflammation. *Pain*.

[20] Wang Y., Liu M., Yang F., Chen H., Wang Y., Liu J. (2024). The Associations of Socioeconomic Status, Social Activities, and Loneliness With Depressive Symptoms in Adults Aged 50 Years and Older Across 24 Countries: Findings From Five Prospective Cohort Studies. *The Lancet Healthy Longevity*.

[21] Bair M. J., Robinson R. L., Katon W., Kroenke K. (2003). Depression and Pain Comorbidity - A Literature Review. *Archives of Internal Medicine*.

[22] Zimmer Z., Fraser K., Grol-Prokopczyk H., Zajacova A. (2022). A Global Study of Pain Prevalence Across 52 Countries: Examining the Role of Country-Level Contextual Factors. *Pain*.

[23] Imai R., Imaoka M., Nakao H. (2020). Association Between Chronic Pain and Pre-Frailty in Japanese Community-Dwelling Older Adults: A Cross-Sectional Study. *PLoS One*.

[24] Saraiva M. D., Suzuki G. S., Lin S. M., de Andrade D. C., Jacob-Filho W., Suemoto C. K. (2018). Persistent Pain is a Risk Factor for Frailty: A Systematic Review and Meta-Analysis From Prospective Longitudinal Studies. *Age and Ageing*.

[25] Lin T., Zhao Y., Xia X., Ge N., Yue J. (2020). Association Between Frailty and Chronic Pain Among Older Adults: A Systematic Review and Meta-Analysis. *European Geriatric Medicine*.

[26] Verbrugge L. M., Jette A. M. (1994). The Disablement Process. *Social Science & Medicine*.

[27] Otones Reyes P., García Perea E., Rico Blázquez M., Pedraz Marcos A. (2020). Prevalence and Correlates of Frailty in Community-Dwelling Older Adults With Chronic Pain: A Cross-Sectional Study. *Pain Management Nursing*.

[28] O’Brien M. S., McDougall J. J. (2019). Age and Frailty as Risk Factors for the Development of Osteoarthritis. *Mechanisms of Ageing and Development*.

[29] Crosignani S., Orlandini L., Baruffi S., Froldi M., Cesari M. (2022). Frailty and Persistent Pain in Oncological Patients Undergoing Rehabilitation. *The Journal of Frailty & Aging*.

[30] O’Caoimh R., Sezgin D., O’Donovan M. R. (2021). Prevalence of Frailty in 62 Countries Across the World: A Systematic Review and Meta-Analysis of Population-Level Studies. *Age and Ageing*.

[31] Liu H., Yang X., Guo L. L. (2022). Frailty and Incident Depressive Symptoms During Short- and Long-Term Follow-Up Period in the Middle-Aged and Elderly: Findings From the Chinese Nationwide Cohort Study. *Frontiers in Psychiatry*.

[32] Mayerl H., Stolz E., Freidl W. (2020). Frailty and Depression: Reciprocal Influences or Common Causes?. *Social Science & Medicine*.

[33] Sang N., Liu R.-C., Zhang M.-H. (2024). Changes in Frailty and Depressive Symptoms Among Middle-Aged and Older Chinese People: A Nationwide Cohort Study. *BMC Public Health*.

[34] Chu W., Chang S. F., Ho H. Y., Lin H. C. (2019). The Relationship Between Depression and Frailty in Community-Dwelling Older People: A Systematic Review and Meta-Analysis of 84,351 Older Adults. *Journal of Nursing Scholarship*.

[35] Prina A. M., Stubbs B., Veronese N. (2019). Depression and Incidence of Frailty in Older People From Six Latin American Countries. *The American Journal of Geriatric Psychiatry*.

[36] Setiati S., Soejono C. H., Harimurti K. (2021). Frailty and Its Associated Risk Factors: First Phase Analysis of Multicentre Indonesia Longitudinal Aging Study. *Frontiers in Medicine*.

[37] Luo X., Ruan Z., Liu L. (2023). Causal Relationship Between Depression and Aging: A Bidirectional Two-Sample Mendelian Randomization Study. *Aging Clinical and Experimental Research*.

[38] Brown P. J., Rutherford B. R., Yaffe K. (2016). The Depressed Frail Phenotype: The Clinical Manifestation of Increased Biological Aging. *The American Journal of Geriatric Psychiatry*.

[39] Rong T., Kang L., Zhang Y., Yin L., Gao Y., Gao J. (2025). A Serial Mediation Model of Chronic Multimorbidity and Frailty in Older Adults: The Role of Pain and Depressive Symptoms. *Psychogeriatrics*.

[40] Milligen B. A. Lever-van, Lamers F., Smit J. H., Penninx B. W. J. H. (2020). Physiological Stress Markers, Mental Health and Objective Physical Function. *Journal of Psychosomatic Research*.

[41] Tian X., Wang C., Qiao X. (2018). Association Between Pain and Frailty Among Chinese Community-Dwelling Older Adults: Depression as a Mediator and Its Interaction With Pain. *Pain*.

[42] Hirase T., Kataoka H., Nakano J., Inokuchi S., Sakamoto J., Okita M. (2018). Impact of Frailty on Chronic Pain, Activities of Daily Living and Physical Activity in Community-Dwelling Older Adults: A Cross-Sectional Study. *Geriatrics & Gerontology International*.

[43] Wang Q., Song D., Lin Q. (2023). The Critical Role of Physical Frailty and Function on Depressive Symptoms Among Community-Dwelling Older Adults in China: A Cross-Sectional Study. *Frontiers in Psychiatry*.

[44] Dai Z., Wu Y., Chen J., Huang S., Zheng H. (2024). Assessment of Relationships Between Frailty and Chronic Pain: A Bidirectional Two-Sample Mendelian Randomisation Study. *Age and Ageing*.

[45] Sang N., Li B. H., Zhang M. Y. (2023). Bidirectional Causal Relationship Between Depression and Frailty: A Univariate and Multivariate Mendelian Randomisation Study. *Age and Ageing*.

[46] Yao C., Zhang Y., Lu P. (2023). Exploring the Bidirectional Relationship Between Pain and Mental Disorders: A Comprehensive Mendelian Randomization Study. *The Journal of Headache and Pain*.

[47] Muhammad T., Rashid M. (2022). Prevalence and Correlates of Pain and Associated Depression Among Community-Dwelling Older Adults: Cross-Sectional Findings From LASI, 2017–2018. *Depression and Anxiety*.

[48] Qing L., Zhu Y., Feng L. (2024). Exploring the Association Between Frailty Index and Low Back Pain in Middle-Aged and Older Chinese Adults: A Cross-Sectional Analysis of Data From the China Health and Retirement Longitudinal Study (CHARLS). *BMJ Open*.

[49] Zhang Y., Yu G., Bai W. (2024). Association of Depression and Sleep Quality With Frailty: A Cross-Sectional Study in China. *Frontiers in Public Health*.

[50] Zhao Y., Hu Y., Smith J. P., Strauss J., Yang G. (2014). Cohort Profile: The China Health and Retirement Longitudinal Study (CHARLS). *International Journal of Epidemiology*.

[51] Mohebbi M., Nguyen V., McNeil J. J. (2018). Psychometric Properties of a Short Form of the Center for Epidemiologic Studies Depression (CES-D-10) Scale for Screening Depressive Symptoms in Healthy Community Dwelling Older Adults. *General Hospital Psychiatry*.

[52] Chen H., Mui A. C. (2014). Factorial Validity of the Center for Epidemiologic Studies Depression Scale Short Form in Older Population in China. *International Psychogeriatrics*.

[53] Meng X., Li D., Wang Y., Han C. (2024). Sleep Duration and Pain During the COVID-19 Pandemic With Depression and Chronic Diseases as Mediators. *Scientific Reports*.

[54] Searle S. D., Mitnitski A., Gahbauer E. A., Gill T. M., Rockwood K. (2008). A Standard Procedure for Creating a Frailty Index. *BMC Geriatrics*.

[55] Man T., Zhao Y., Mai H., Bian Y. (2024). The Influence of Middle-Aged and Older Adults’ Social Capital and Education on Physical Function: Evidence From the China Health and Retirement Longitudinal Study. *Frontiers in Public Health*.

[56] Qiu Y., Ma Y., Huang X. (2022). Bidirectional Relationship Between Body Pain and Depressive Symptoms: A Pooled Analysis of Two National Aging Cohort Studies. *Frontiers in Psychiatry*.

[57] Sun Y., Li X., Liu H. (2024). Predictive Role of Depressive Symptoms on Frailty and Its Components in Chinese Middle-Aged and Older Adults: A Longitudinal Analysis. *BMC Public Health*.

[58] Li J., Guo S., Ma R. (2024). Comparison of the Effects of Imputation Methods for Missing Data in Predictive Modelling of Cohort Study Datasets. *BMC Medical Research Methodology*.

[59] Hamaker E. L. (2023). The Within-Between Dispute in Cross-Lagged Panel Research and How to Move Forward. *Psychological Methods*.

[60] Usami S. (2021). On the Differences Between General Cross-Lagged Panel Model and Random-Intercept Cross-Lagged Panel Model: Interpretation of Cross-Lagged Parameters and Model Choice. *Structural Equation Modeling: A Multidisciplinary Journal*.

[61] Mund M., Johnson M. D., Nestler S. (2021). Changes in Size and Interpretation of Parameter Estimates in Within-Person Models in the Presence of Time-Invariant and Time-Varying Covariates. *Frontiers in Psychology*.

[62] Orth U., Clark D. A., Donnellan M. B., Robins R. W. (2021). Testing Prospective Effects in Longitudinal Research: Comparing Seven Competing Cross-Lagged Models. *Journal of Personality and Social Psychology*.

[63] Little T. D., Preacher K. J., Selig J. P., Card N. A. (2007). New Developments in Latent Variable Panel Analyses of Longitudinal Data. *International Journal of Behavioral Development*.

[64] Gove W. R., Ortega S. T., Style C. B. (1989). The Maturational and Role Perspectives on Aging and Self Through the Adult Years: An Empirical Evaluation. *American Journal of Sociology*.

[65] Wirth M., Voss A., Rothermund K. (2023). Age Differences in Everyday Emotional Experience: Testing Core Predictions of Socioemotional Selectivity Theory With the MIVA Model. *The Journals of Gerontology: Series B*.

[66] Funkhouser C. J., Kaiser A. J. E., Alqueza K. L. (2021). Depression Risk Factors and Affect Dynamics: An Experience Sampling Study. *Journal of Psychiatric Research*.

[67] Katana M., Röcke C., Allemand M. (2020). Intra- and Interindividual Differences in the Within-Person Coupling Between Daily Pain and Affect of Older Adults. *Journal of Behavioral Medicine*.

[68] Nerurkar L., Siebert S., McInnes I. B., Cavanagh J. (2019). Rheumatoid Arthritis and Depression: An Inflammatory Perspective. *The Lancet Psychiatry*.

[69] Suneson K., Lindahl J., Chamli Hårsmar S., Söderberg G., Lindqvist D. (2021). Inflammatory Depression-Mechanisms and Non-Pharmacological Interventions. *International Journal of Molecular Sciences*.

[70] Ferrucci L., Fabbri E. (2018). Inflammageing: Chronic Inflammation in Ageing, Cardiovascular Disease, and Frailty. *Nature Reviews Cardiology*.

[71] Chen G., Zhang Y.-Q., Qadri Y. J., Serhan C. N., Ji R.-R. (2018). Microglia in Pain: Detrimental and Protective Roles in Pathogenesis and Resolution of Pain. *Neuron*.

[72] Franceschi C., Bonafè M., Valensin S. (2000). Inflamm-Aging: An Evolutionary Perspective on Immunosenescence. *Annals of the New York Academy of Sciences*.

[73] Bergens O., Nilsson A., Papaioannou K.-G., Kadi F. (2021). Sedentary Patterns and Systemic Inflammation: Sex-Specific Links in Older Adults. *Frontiers in Physiology*.

[74] Harsanyi S., Kupcova I., Danisovic L., Klein M. (2023). Selected Biomarkers of Depression: What are the Effects of Cytokines and Inflammation?. *International Journal of Molecular Sciences*.

[75] Kato S., Demura S., Shinmura K. (2021). Association of Low Back Pain With Muscle Weakness, Decreased Mobility Function, and Malnutrition in Older Women: A Cross-Sectional Study. *PLoS One*.

[76] Taylor J. L., Parker L. J., Szanton S. L., Thorpe R. J. (2018). The Association of Pain, Race and Slow Gait Speed in Older Adults. *Geriatric Nursing*.

[77] Karp J. F., Shega J. W., Morone N. E., Weiner D. K. (2008). Advances in Understanding the Mechanisms and Management of Persistent Pain in Older Adults. *British Journal of Anaesthesia*.

[78] Cui L., Li S., Wang S. (2024). Major Depressive Disorder: Hypothesis, Mechanism, Prevention and Treatment. *Signal Transduction and Targeted Therapy*.

[79] Dong L., Xie Y., Zou X. (2022). Association Between Sleep Duration and Depression in US Adults: A Cross-Sectional Study. *Journal of Affective Disorders*.

[80] Soini E., Rosenström T., Määttänen I., Jokela M. (2024). Physical Activity and Specific Symptoms of Depression: A Pooled Analysis of Six Cohort Studies. *Journal of Affective Disorders*.

[81] Gao Q., Hu K., Yan C. (2021). Associated Factors of Sarcopenia in Community-Dwelling Older Adults: A Systematic Review and Meta-Analysis. *Nutrients*.

[82] Wu Q., Liu B., Tonmoy S. (2018). Depression and Risk of Fracture and Bone Loss: An Updated Meta-Analysis of Prospective Studies. *Osteoporosis International*.

[83] Robles B., Jewell M. P., Thomas Tobin C. S., Smith L. V., Kuo T. (2021). Varying Levels of Depressive Symptoms and Lifestyle Health Behaviors in a Low Income, Urban Population. *Journal of Behavioral Medicine*.

[84] Rush A. J., Thase M. E. (2018). Improving Depression Outcome by Patient-Centered Medical Management. *American Journal of Psychiatry*.

[85] Beck A. T. (2008). The Evolution of the Cognitive Model of Depression and Its Neurobiological Correlates. *American Journal of Psychiatry*.

[86] Naushad N., Dunn L. B., Muñoz R. F., Leykin Y. (2018). Depression Increases Subjective Stigma of Chronic Pain. *Journal of Affective Disorders*.

[87] Brown P. J., Ciarleglio A., Roose S. P. (2021). Frailty Worsens Antidepressant Treatment Outcomes in Late Life Depression. *The American Journal of Geriatric Psychiatry*.

[88] Brown P. J., Ciarleglio A., Roose S. P. (2022). Frailty and Depression in Late Life: A High-Risk Comorbidity With Distinctive Clinical Presentation and Poor Antidepressant Response. *The Journals of Gerontology: Series A*.

[89] Xiao H., Zhu W., Jing D. (2025). Association Between Frailty and Common Psychiatric Disorders: A Bidirectional Mendelian Randomization Study. *Journal of Affective Disorders*.

[90] Belloni G., Cesari M. (2019). Frailty and Intrinsic Capacity: Two Distinct but Related Constructs. *Frontiers in Medicine*.

[91] Novic A. J., Seib C., Burton N. W. (2022). Longitudinal Association of Physical Activity, Mastery and Psychological Distress in Mid-Aged Adults Over 9-Years. *International Journal of Environmental Research and Public Health*.

[92] Schrempft S., Jackowska M., Hamer M., Steptoe A. (2019). Associations Between Social Isolation, Loneliness, and Objective Physical Activity in Older Men and Women. *BMC Public Health*.

[93] Jiang R., Noble S., Rosenblatt M. (2024). The Brain Structure, Inflammatory, and Genetic Mechanisms Mediate the Association Between Physical Frailty and Depression. *Nature Communications*.

[94] Bakshi N., Hart A. L., Lee M. C. (2021). Chronic Pain in Patients With Inflammatory Bowel Disease. *Pain*.

[95] Koncicki H. M., Unruh M., Schell J. O. (2017). Pain Management in CKD: A Guide for Nephrology Providers. *American Journal of Kidney Diseases*.

[96] Huang H., Ni L., Zhang L., Zhou J., Peng B. (2025). Longitudinal Association Between Frailty and Pain in Three Prospective Cohorts of Older Population. *The Journal of Nutrition, Health and Aging*.

[97] Mancini F. (2013). Focus on Pain in the Blind. *Pain*.

[98] Mansutti I., Tomé-Pires C., Chiappinotto S., Palese A. (2023). Facilitating Pain Assessment and Communication in People With Deafness: A Systematic Review. *BMC Public Health*.

[99] Brunes A., Heir T. (2020). Social Interactions, Experiences With Adverse Life Events and Depressive Symptoms in Individuals With Visual Impairment: A Cross-Sectional Study. *BMC Psychiatry*.

[100] Tan Y., Fang L., Zhu Y., Hashimoto K. (2024). Relationship Between Hearing Loss and Depression: A Cross-Sectional Analysis From the National Health and Nutrition Examination Survey 2015–2018. *Journal of Psychiatric Research*.

[101] Yuan Y., Peng C., Burr J. A., Lapane K. L. (2023). Frailty, Cognitive Impairment, and Depressive Symptoms in Chinese Older Adults: An 8-Year Multi-Trajectory Analysis. *BMC Geriatrics*.

[102] Rodríguez-Laso Á., García-García F. J., Rodríguez-Mañas L. (2022). Transitions Between Frailty States and Its Predictors in a Cohort of Community-Dwelling Spaniards. *Journal of the American Medical Directors Association*.

[103] Rohrer J. M., Murayama K. (2023). These are not the Effects You are Looking for: Causality and the Within-/Between-Persons Distinction in Longitudinal Data Analysis. *Advances in Methods and Practices in Psychological Science*.

[104] Ahrend J. N., Jobski K., Bantel C., Hoffmann F. (2025). Pain Intensity and Comorbid Depressive Symptoms in the General Population: An Analysis of the German Health Update Study (GEDA 2019/2020-EHIS). *European Journal of Pain*.

[105] Wong J. J., Tricco A. C., Côté P. (2022). Association Between Depressive Symptoms or Depression and Health Outcomes for Low Back Pain: A Systematic Review and Meta-Analysis. *Journal of General Internal Medicine*.

[106] Jiang L., Sheng Y., Li J., Chen J., Xue K., Kong Q. (2024). Association Between Pain Intensity and Depressive Status in Patients With Hip Fracture: An Observational Study. *Medicine (Baltimore)*.

[107] Hider S. L., Whitehurst D. G., Thomas E., Foster N. E. (2015). Pain Location Matters: The Impact of Leg Pain on Health Care use, Work Disability and Quality of Life in Patients With Low Back Pain. *European Spine Journal*.

[108] Jha M. K., Greer T. L., Grannemann B. D., Carmody T., Rush A. J., Trivedi M. H. (2016). Early Normalization of Quality of Life Predicts Later Remission in Depression: Findings From the CO-MED Trial. *Journal of Affective Disorders*.

[109] Baune B. T., Caniato R. N., Garcia-Alcaraz M. A., Berger K. (2008). Combined Effects of Major Depression, Pain and Somatic Disorders on General Functioning in the General Adult Population. *Pain*.

[110] Shin J. H., Kang G. A., Kim S. Y., Won W. C., Yoon J. Y. (2024). Bidirectional Relationship Between Depression and Frailty in Older Adults Aged 70-84 Years Using Random Intercepts Cross-Lagged Panel Analysis. *Research in Community and Public Health Nursing*.

[111] Panza F., Solfrizzi V., Sardone R. (2023). Depressive and Biopsychosocial Frailty Phenotypes: Impact on Late-Life Cognitive Disorders. *Journal of Alzheimer’s Disease*.

[112] Ye B., Li Y., Bao Z., Gao J. (2024). Psychological Resilience and Frailty Progression in Older Adults. *JAMA Network Open*.

